# Risk factors for human brucellosis among a pastoralist community in South-West Kenya, 2015

**DOI:** 10.1186/s13104-018-3961-x

**Published:** 2018-12-05

**Authors:** Mathew Muturi, Austine Bitek, Athman Mwatondo, Eric Osoro, Doris Marwanga, Zeinab Gura, Phillip Ngere, Zipporah Nganga, S. M. Thumbi, Kariuki Njenga

**Affiliations:** 1 Kenya Zoonotic Disease Unit–Ministry of Agriculture, Livestock and Fisheries and Ministry of Health, P.O. Box 20811-00202, Nairobi, Kenya; 2 Food and Agriculture Organization of the United Nations, Nairobi, Kenya; 3 Paul G. Allen School for Global Animal Health, Washington State University, Pullman, WA USA; 4 Kenya Medical Research Institute, Nairobi, Kenya; 5 Kenya Field Epidemiology and Laboratory Training Program, Nairobi, Kenya; 6 County Government of Kajiado, Kajiado, Kenya; 7 Jomo Kenyatta University of Agriculture and Technology, Nairobi, Kenya

**Keywords:** Brucellosis, Risk factors, Kenya

## Abstract

**Objective:**

Brucellosis is one of the top five priority zoonosis in Kenya because of the socio-economic burden of the disease, especially among traditional, livestock keeping communities. We conducted a 1 year, hospital based, unmatched case–control study to determine risk factors for brucellosis among Maasai pastoralists of Kajiado County in 2016. A case was defined by a clinical criteria; fever or history of fever and two clinical signs suggestive of brucellosis and a positive competitive enzyme-linked immunosorbent assay test (c-ELISA). A control was defined as patients visiting the study facility with negative c-ELISA. Unconditional logistic regression was used to study association between exposure variables and brucellosis using odds ratios (OR) and 95% confidence intervals (CI).

**Results:**

Forty-three cases and 86 controls were recruited from a population of 4792 individuals in 801 households. The mean age for the cases was 48.7 years while that of the controls was 37.6 years. The dominant gender for both cases (62.7%) and controls (58.1%) groups was female. Regular consumption of un-boiled raw milk and assisting animals in delivery were significantly associated with brucellosis by OR 7.7 (95% CI 1.5–40.1) and OR 3.7 (95% CI 1.1–13.5), respectively.

## Introduction

Brucellosis is a debilitating febrile illness in humans and reproductive disease of livestock, caused by bacteria of the genus *Brucella* [1]. There are six *Brucella* species based on primary host preference, but only four have zoonotic potential; *B. melitensis* (goats and sheep), *Brucella abortus* (cattle), *B. suis* (swine) and *B. canis* (dogs) [2–5]. Human infection occurs through direct contact with infected animal tissues like products of abortion and blood or ingestion of unpasteurized milk and dairy products [2, 6]. Although livestock are the primary source of human infection, wild animals may act as reservoirs in regions with human-wildlife interaction [7, 8]. Human brucellosis presents as an acute to chronic illness characterized by fever and other constitutional symptoms such as joint pains, fatigue and muscle ache that vary with the stage of infection and body system affected [9, 10]. The disease has a low mortality rate, but the relapsing and chronic nature of human infection, the long cause of treatment and negative implication on livestock trade qualifies brucellosis as a serious public health and socio-economic problem [2, 9, 11–15].

Brucellosis is the most common zoonotic infection globally with more than half a million human cases annually, however, infection rates vary significantly between developed and developing countries [1, 16, 17]. The human disease has been eliminated in most developed countries like Canada, Japan and Australia but remains endemic in most developing countries in Asia, the Middle East, Eastern Europe, Latin America and Africa [1, 16, 18–20].

In Kenya, brucellosis is ranked as a top priority zoonosis due to the socio-economic burden and amenability to control, however, as is common with other neglected zoonotic diseases, establishing the true morbidity and socio-economic impact of the disease is a challenge because of misdiagnosis and underreporting [21]. Studies in Kenya indicate high prevalence in humans and livestock although this varies with geographical region and livestock production system [22–27]. Brucellosis is endemic in Kenya and identifying potential risk factors of brucellosis among the most vulnerable populations; primarily rural livestock keeping communities is important in defining control and prevention strategies. We conducted a case–control study in a pastoral community in rural Kenya to identify potential risk factors for brucellosis as a step towards comprehensive understanding of the disease among pastoralists to inform public health interventions.

## Main text

### Materials and methods

#### Study area and population

**Fig. 1:**
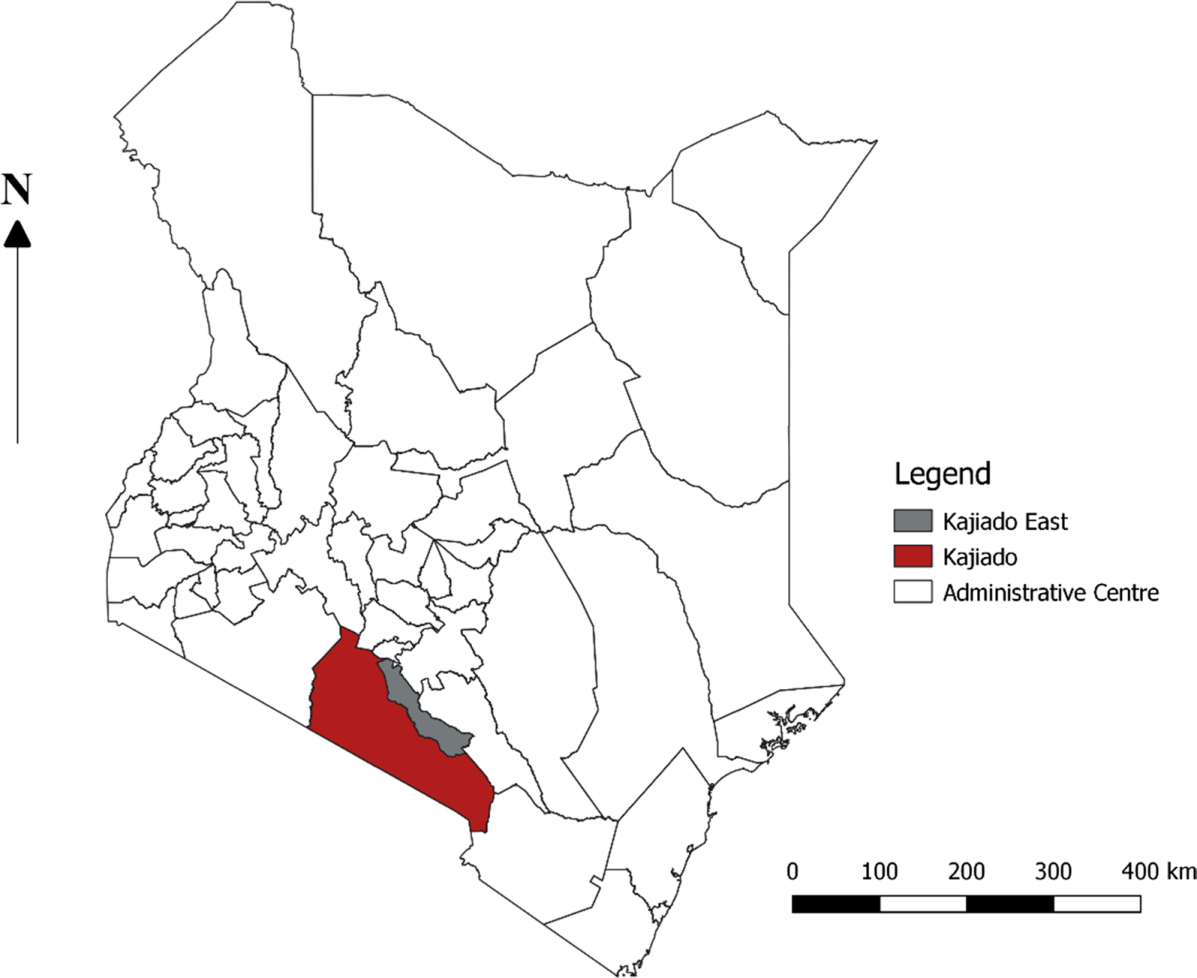
Map of Kenya showing Kajiado County in red and the study site in grey

The study was conducted in Arroi, Sultan-Hamud and Mashuru sub-counties in Kajiado East sub-county, Kenya (Fig. 1). The study area is an arid rangeland inhabited primarily by the Maasai nomadic pastoralist community [23, 28]. The site was selected because a previous study had reported high brucellosis prevalence and because it represent an ecosystem with high frequency of human-livestock-wildlife interaction [23, 29, 30].

#### Study design

We conducted a hospital based unmatched case–control study in three health facilities that historically had the highest patient load in the year preceding the study. Participants were recruited from 80 randomly selected households in the study area that were part of an ongoing longitudinal brucellosis study in humans and livestock (population = 4792 people). To enhance case finding at health facilities, recruited household members were sensitized on brucellosis using a community level case definition adapted from the World Health Organisation, and provided with free treatment at the participating health facility [2]. The community case definition for brucellosis used was fever of undetermined origin with at least one of the following symptoms; chills, lethargy, joint pains, body ache, abdominal pain and headaches.

#### Sample size calculation

Sample size was calculated using the Kelsey Kelsey formula for unmatched case control studies using an open-Epi version 2 open source online calculator (http://www.openepi.com) [31]. The appropriate sample size was determined using a power of 0.8 and significance level of 0.05 to detect an odds ratio greater than 3 for exposure factors present in 20% of controls as estimated in other similar studies [3, 32]. A control to case ratio of 2:1 was used to improve study power. This yielded a sample size of 43 cases and 86 controls.

#### Selection of cases and controls

A case was defined as any person from the study population presenting to any of the three health facilities with fever or history of fever (> 37.5 °C) and at-least two of the following signs; joint pains, joint swelling, headache, backache and was negative for malaria and salmonellosis on rapid diagnostic tests and with a positive c-ELISA Immunoglobulin M (IgM) or Immunoglobulin G (IgG) result. A control was defined as a person from the same study population presenting to the study facilities with history of fever within the same study period and was negative for brucellosis by c-ELISA IgM and IgG. Cases were tested for malaria and Salmonellosis because the diseases are common aetiologies of similar clinical disease.

#### Laboratory testing

Laboratory testing was carried out at the Kenya Medical Research Institute using IgM and IgG ELISA kit sourced from Immuno-Biological Laboratories, America (Minneapolis, Minnesota). All assays were conducted as per manufacturer’s instructions. Briefly, human sera were diluted at 1:10 with sample diluent, added to microtitre plates pre-coated with *Brucella* antigen (*Brucella abortus*, strain W99; lysate of a NaCl extract) and incubated at room temperature for 1 h. Conjugate was added and incubated for 30 min before adding substrate. The conjugate–substrate reaction was terminated after 20 min by adding a stop solution. Sample optical densities (ODs) were read at 450 nm. Equivocal samples were not included in analysis.

#### Questionnaire and interviewing

A study nurse was stationed in each of the three facilities. Once a patient was identified as a member of the study population during triage (coming from a study household), they were directed to the study nurse who examined them and administered a standard questionnaire pre-loaded on a personal data assistant. The questionnaire collected information on patients’ demographic, risk factors, history of illness and point of care test results. Informed consent was obtained from all study participants.

#### Data analysis

A number of risk factors were investigated including consumption of goats, sheep, or cow milk, drinking fresh livestock blood, livestock ownership, herding and slaughtering animals, handling skins and hides, and helping in animal delivery. Bivariate analysis was performed using the Chi squared test. Variables with a p-value ≤ 0.10 in the bivariate analysis were included in a multivariate logistic regression model. Adjusted odds ratios and the corresponding 95% confidence intervals along with the p-values were reported with significance level being set at 5%. Multivariate logistic regression was used to identify risk factors associated with brucellosis and to estimate the magnitude of the adjusted odds ratios (aORs) for each factor while controlling for other confounding factors. Only the significant variables were included in the model to control for confounding and get a final logistic regression model. Only those variables that had a p-value < 0.05 in the final model were considered statistically significant. Data were analyzed using Statistical Analysis Software (SAS) version 9.2.

### Results

#### Patient socio-demographic characteristics

Of the 236 participants from the study population who met the inclusion criteria, majority, 64% were majority female. Participants had a mean age of 40 years (standard deviation = 16.9, range 7–75) and 129 (54.6%) of them were enrolled in the case control study, including 43 cases and 86 controls. The mean age for the cases was 48.7 (standard deviation = 20, range = 10–85) years while that of the controls was 37.6 (standard deviation = 18.8, range = 8–72). Among cases, 70% (n = 30) were between 20 and 59 years. The dominant gender for both cases (62.7%) and controls (58.1%) was female. Majority of both cases and controls were non-skilled laborers and there was no significant difference in socio-demographic characteristics (sex, religion, occupation, marital status and education) between cases and controls besides age.

#### Clinical information

Sixty percent of the cases presented at-least 7 days after the onset of the first symptom while 37% presented between 11 and 60 days after onset of symptoms. The mean number of days between onset of symptoms and visit to hospital was 12 days (standard deviation = 13.3). The most commonly reported symptoms by both cases were headache (83.7%) back pains (62.8%) and joint pains (60.6%). This was similar to the symptoms reported by the controls; headache (82.6%), back pains (47.7%) and joint pains (69.8%).

#### Bivariate analysis

On bivariate analysis, consuming un-boiled cow milk, drinking fresh blood, slaughtering animals (cattle, wild animals), assisting goats in giving birth, handling animal hides were associated with increased risk of brucellosis (*p*-value ≤ 0.1). Of these factors, handling skins and hides, assisting goats with delivery, and consuming un-boiled goat milk were significantly associated with disease (*p*-value ≤ 0.05). Having cattle in the household was found to be protective as shown in the Table 1.

**Table 1:** Bivariate analysis of risk factors for human brucellosis

Variable	Controls (n = 86)	Cases (n = 43)	Crude OR (95% CI)	p-value
	Yes	Yes		
Consume fresh goat milk				
More than 3 times a week	14	14	2.4 (1.0–6.0)	0.114
Less than 3 times a week	21	8	0.9 (0.4–2.4)	
No	51	21	1.0	
Consume cow milk				
Boiled	82	32	7.7 (1.5–40.1)	0.016
Unboiled	2	6		
Consume fresh sheep milk				
More than 3 times a week	1	1	2.1 (0.1–34.1)	0.756
Less than 3 times a week	4	3	1.6 (0.3–7.3)	
No	81	39	1.0	
Drink fresh blood				
Yes	6	7	2.6 (0.8–8.3)	0.098
No	80	36		
Had cattle in the household				
Yes	55	26	0.1 (0.0–0.9)	0.035
No	31	17		
Slaughter cattle at home				
Occasionally	54	32	2.3 (0.8–6.2)	0.102
Never	23	6		
Herding sheep				
Several times a week	16	14	2.0 (0.5–7.8)	0.196
Occasionally	49	19	0.9 (0.2–3.2)	
Never	9	4	1.0	
Assisting sheep in delivery				
Several times a week	1	1	4.0 (0.2–72.2)	0.116
Occasionally	45	30	2.7 (1.0–6.9)	
Never	28	7	1.0	
Slaughtering goats at home				
Several times a week	1	1	4.8 (0.3–90.3)	0.115
Occasionally	53	33	3.0 (1.0–8.6)	
Never	24	5	1.0	
Assisting goats in delivery				
Occasionally	48	31	3.7 (1.3–10.7)	0.043
Never	29	5	1.0	
Slaughtering wild animals				
Yes	1	3		0.073
No	82	40	6.4 (0.6–63.2)	
Cleaning animal barns				
Several times a week	57	5	0.4 (0.1–1.3)	0.132
Occasionally	19	14		
Handle animal hides				
Yes	30	23	2.1 (1.2–4.5)	0.043
No	56	20		

#### Multivariable analysis results

On multivariate logistic regression analysis consuming un-boiled cow milk (OR 7.7, 95% CI 1.5–40.1) and assisting animals in delivery (OR 3.7, 95% CI 1.1–13.5) remained significantly associated with brucellosis as shown in Table 2.

**Table 2:** Multivariate logistic regression of factors associated with brucellosis

Variable	Adjusted OR (95% CI)	p-value
Slaughter animals	6.2 (1.1–34.7)	0.350
Handling animal hides	1.3 (0.5–3.6)	0.563
Own cattle	0.6 (0.2–1.6)	0.327
Drinks fresh blood	3.1 (0.8–11.2)	0.088
Assisting livestock in delivery	*3.7 (1.1–13.5)*	*0.050*
Drinking un-boiled cow milk	*7.7 (1.5–40.1)*	*0.036*

### Discussion

Our case–control study identified consumption of raw cow milk, assisting livestock in delivery, and handling animal hides as risk factors on bivariate analysis. However, only assisting livestock in delivery and drinking un-boiled cow milk remained significant risk facts after multivariate analysis. The association between assisting animals with delivery and increased risk of infection has been reported in other studies carried out in similar settings in East Africa [23, 33] Chad [34], the Middle East [35] and in Turkey [36, 37]. Given that *Brucella* spp. are known to have a predilection for reproductive organs particularly placenta and aborted fetuses, it is logical that assisting animals in delivery increases risk of infection [23]. The risk of brucellosis associated with consumption of un-boiled milk has been well documented [22, 23, 38]. Interestingly, even though most of the pastoralists around the world know about this risk, majority of them still consume raw milk as a tradition and for cultural reasons [39]. Although opinion differs between authors on whether direct contact with livestock (assisting in delivery, milking and feeding) or indirect contact with livestock (consumption of animal products) is a stronger risk factor, we found greater association with disease from consuming animal products than direct contact with animal. This finding is in agreement with other studies carried out within the East Africa region [23, 40, 41]. Studies have shown that consumption of unpasteurized milk is a common practise in Kenya, including communities in urban areas such as where 77% of households reported the risky practice [42]. Some studies show education and occupation are significant risk factors contrary to our data that shows there was no significant difference on the two variables between cases and controls. A possible explanation is the study area is a rural, predominantly Maasai agro-pastoral community where most households practise a traditional livestock rearing lifestyle. This means that cases and controls have similar occupation and education levels.

### Conclusion and recommendations

The findings of this study show a significant association between infection and consumption of unpasteurized milk and assisting animals with delivery. This findings show that animal handlers; primarily farmers and animal health workers and people who consume unpasteurized milk; a common practise in Kenya, are at the greatest risk. We recommend Public health education on brucellosis transmission and prevention, specifically use of protective personal equipment when assisting animals in delivery and boiling of milk should be offered to farmers and the general public, respectively.

## Limitations

There were some limitations to the study. Case–control studies are prone to selection bias but we took measures to minimise the same; we recruited cases and controls from households participating in an ongoing cohort study of brucellosis in livestock. This meant cases and controls were recruited from households with similar characteristics, which in turn minimises selection bias. Another significant limitation is the limited sample size. The study only recruited cases and controls from an ongoing study that had recruited 810 households with 4792 people; this limited the number of study participants who could be included in our analysis.

## Abbreviations

AOR: adjusted odds ratio
C-ELISA: competitive enzyme-linked immunosorbent assay
CI: confidence interval
IgM: immunoglobulin M
IgG: immunoglobulin G
OD: optical density
OR: odds ration
